# Associations of socio-demographic characteristics, well-being, school absenteeism, and substance use with recreational nitrous oxide use among adolescents: A cross-sectional study

**DOI:** 10.1371/journal.pone.0247230

**Published:** 2021-02-18

**Authors:** Suzanne J. van den Toren, Amy van Grieken, Hein Raat

**Affiliations:** Department of Public Health, Erasmus University Medical Center, Rotterdam, the Netherlands; Chiba Daigaku, JAPAN

## Abstract

**Purpose:**

A rapid increase of recreational nitrous oxide use (i.e. laughing gas, N_2_O) has been reported in several countries, while it has received limited attention in scientific research. We aimed to study the association of socio-demographic characteristics, mental well-being, sickness absence, truancy, and substance use with the frequency of lifetime nitrous oxide use among adolescents.

**Methods:**

We used self-reported questionnaire data of adolescents (N = 555) attending secondary schools to cross-sectionally assess the frequency of nitrous oxide use and potential factors associated with nitrous oxide use, such as gender, mental well-being, and binge drinking. Ordinal logistic regression models were applied with lifetime nitrous oxide use (never, once, ≥ two times) as the outcome variable.

**Results:**

Adolescents were on average 15.6 years old (SD = 0.83, range 14–18), 47.0% were female. In total, 86 (15.6%) adolescents had used nitrous oxide at least once in their life.

In the multivariable ordinal regression model, the risk of having a higher category of lifetime nitrous oxide use was associated with a non-Dutch ethnic background (OR = 2.10, 95% CI 1.22; 3.61), attending pre-vocational education (OR = 1.88, 95% CI 1.06; 3.34), a higher score on the scale of externalizing problems (OR = 1.10, 95% CI 1.01; 1.20), binge drinking twice or more in the past four weeks (OR = 2.49, 95% CI 1.25; 4.94), and cannabis use (OR = 1.98, 95% CI 1.03; 3.79).

**Conclusions:**

Youth Health Care professionals should be aware of nitrous oxide use in adolescents, especially among adolescents with a non-Dutch ethnic background, lower education levels, externalizing problems, frequent binge drinking, and cannabis use.

## Introduction

The recreational use of nitrous oxide (i.e. laughing gas, N_2_O) has been reported to increase rapidly in western countries [1–4]. In a study among night lifers in 2016, nitrous oxide has the third-highest percentage of lifetime (53.5%) and past-year ‘drug users’ (37.3%), after cannabis and ecstasy [2].

Studies using a representative sample of 12-16-year-old secondary school students demonstrated an increase in lifetime nitrous oxide use between 2015 (7.8%) and 2017 (9.4%), which was significant for girls [3,4].

Between the ages of 12 and 16 years, the lifetime use of nitrous oxide was found to increase from 3.5% to 16.9%, with the biggest increase at 14 and 15 years-of-age [4]. Especially in the developing brain of adolescents, the use of nitrous oxide might affect functioning in the long term, as is seen in substance use other than nitrous oxide [5,6].

Recreational nitrous oxide use is mostly used by inhaling the nitrous oxide from balloons, these balloons are filled with nitrous oxide through cylinders or whipped cream dispensers [7]. At the time of this study, a cylinder or whipped cream dispenser could be legally obtained under the Dutch Commodities Act [8]. In rare cases, users inhale the gas directly from the cylinder or whipped cream dispenser, which could result in complications, such as a frostbite injury [9]. The use of nitrous oxide causes lowered consciousness, dizziness, and deformation of vision and sound. These consequences may result in euphoric feelings as well as anxiety or distress. The effects disappear circa five minutes after inhaling, but there is some evidence that the effects may linger on for hours [7].

Recently, awareness of the risks of recreational nitrous oxide use is growing [1,10–12]. In the short term, excessive nitrous oxide use at one occasion might cause oxygen deficiency in the brain. This could lead to dizziness and risk of accidents, for example through falling. Of respondents who lost consciousness after substance use, 11% reported having used nitrous oxide before their black-out [2]. The Dutch police reported an increase in traffic incidents related to the use of nitrous oxide between 2018 and 2019 [11]. In the long term or with excessive use over time, users reported confusion and headache, probably as a result of oxygen deficiency. Also, a deprivation of vitamin B12 could occur. This deprivation may lead to neurological deficits and anemia [1,7,10,12,13]. A study found psychiatric symptoms, such as panic attacks, confusion, or delusions as a result of nitrous oxide abuse in 11 cases out of 91 in total [14].

Substance use, such as alcohol drinking and alcohol intoxication, has been associated with conduct problems and mental health problems among adolescents [15–17]. Furthermore, the onset of alcohol, tobacco, and marijuana use has been associated with school attendance problems, such as truancy [18,19]. So far, it is unknown whether the use of nitrous oxide is associated with similar issues among adolescents.

We aim to explore the association of socio-demographic characteristics, internalizing and externalizing problems, mental well-being, sickness absence from school, truancy, and substance use (tobacco, alcohol, and cannabis) with the frequency of lifetime nitrous oxide use among adolescents in a general population sample.

## Materials and methods

### Study design, setting, and participants

A cross-sectional design was used to explore what factors are associated with lifetime nitrous oxide use among adolescents. Questionnaire data were used derived from the study on the extension of preventive Youth Health Care for adolescents. This extension of preventive health care for adolescents aims to promote health and health behaviors in adolescents above the age of 13 years, with a specific focus on preventive education on substance use and lifestyle [20]. It is offered by the Dutch Youth Health Care in collaboration with the local municipality and schools. The Youth Health Care offers nationwide anticipatory guidance for children and youth to promote growth, development, and health. This guidance generally entails health consultations, which often take place at school with a youth physician or nurse [21]. A project was set up to evaluate the extension of preventive Youth Health Care for adolescents, e.g. by conducting a questionnaire and by organizing focus group interviews among adolescents [22]. For the questionnaire, a power calculation was performed to calculate the target sample size to obtain a statistically significant small to medium effect size of the extension of preventive Youth Health Care for adolescents when compared to a control condition. We assumed an alpha of 0.05 and a power of 0.8, which led to a target sample size of 154 participants per condition.

**Fig 1:**
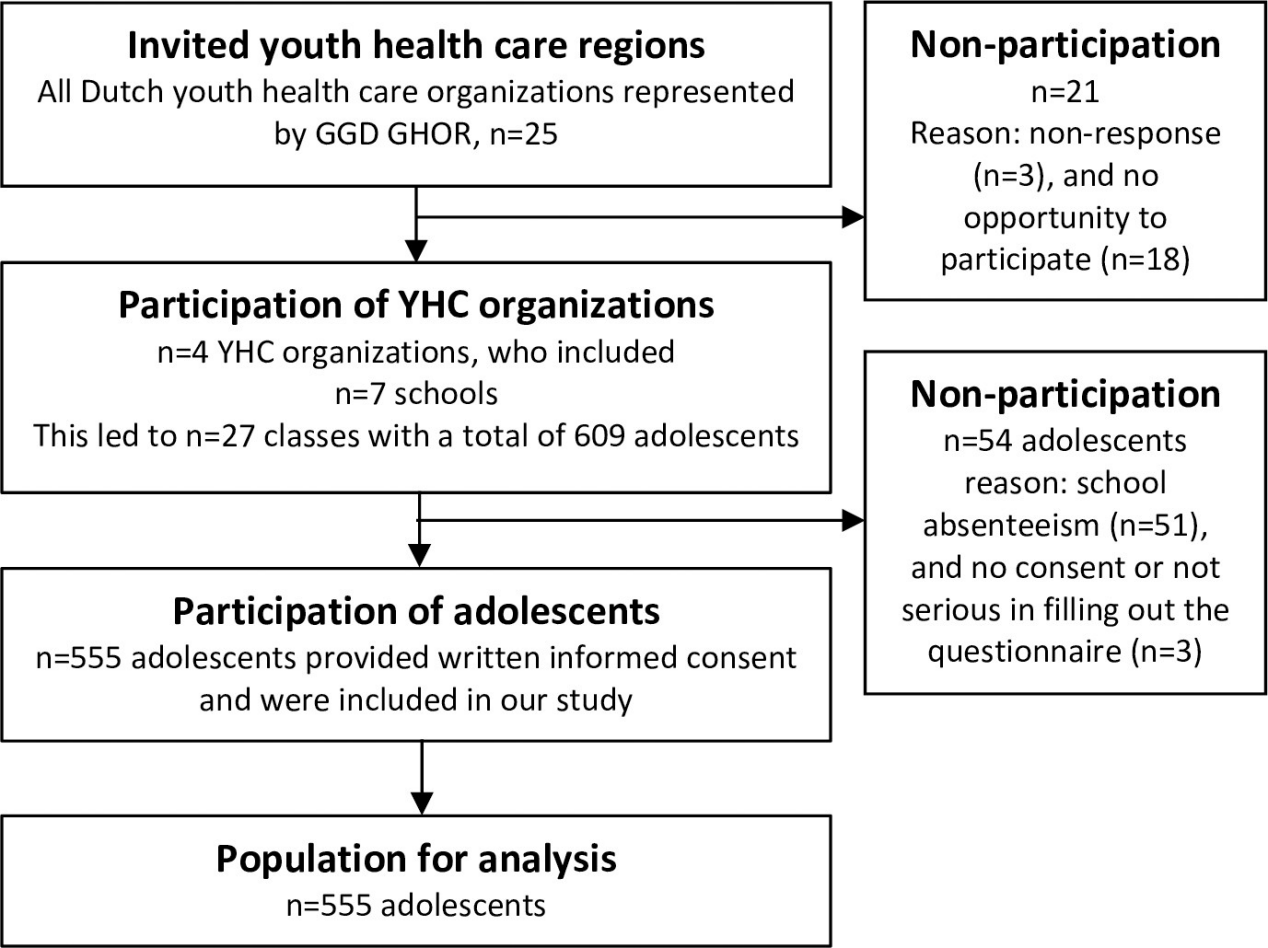
Flowchart of the study population.

All twenty-five organizations in the Dutch Youth Health Care regions were invited to participate in this study (Fig 1). Twenty-two organizations responded to this invitation (88%), of which twelve organizations indicated their willingness to participate (48% of all invited organizations). Four Youth Health Care organizations in four different regions participated in the questionnaire part of the study (other organizations participated in other parts of the project, such as focus groups). Each of these four participating organizations provided a contact person at one or two schools within their region to inform about the possibility of conducting a study at that school. Finally, seven schools within the four different regions were included, which entailed twenty-seven classes. These classes consisted of 609 adolescents in total, of whom 555 adolescents (91.1%) provided informed consent and participated in our study (Fig 1). The contact person at the school was informed about the procedure of preparation and execution of the study. All parents and potential participants (adolescents) were informed prior to the study about the purpose and the procedure of the study; passive consent processes were adopted in relation to parental consent (as approved by the medical ethics committee). Adolescents provided written informed consent before completing the questionnaire (N = 555). The voluntary nature of the research study was stressed in the student information letter, and again at the time of completing the questionnaire. An appointed fieldworker conducted the anonymous online questionnaire in class (+/- 20 minutes) during school hours. Data were collected in the fall of 2016.

The Medical Ethics Committee of the Erasmus University Medical Centre Rotterdam issued a declaration of no objection to conducting this study and permitted to submit the results for publication in a scientific journal (number MEC-2016-297).

### Measurements

The questionnaire assessed nitrous oxide use, socio-demographic characteristics, mental health, sickness absence, truancy, and substance use (see S1 Table for an overview of the questionnaire).

#### Nitrous oxide use

Nitrous oxide use was assessed by the question: “How many times did you use nitrous oxide in your life”. Answer options ranged from ‘never’, ‘once’, ‘twice’, ‘3 times’, ‘4–10 times’, and ‘≥ 11 times’. For analysis purposes, this variable was recoded into three categories: ‘never used’, ‘used one time’, and ‘used ≥ two times’ [23].

Participants who indicated that they had never used nitrous oxide answered a subsequent question: “Do you think you will ever use nitrous oxide?”. Answer options were a 5-point Likert scale ‘definitely’, ‘maybe’, ‘probably not’, ‘definitely not’, and ‘I do not know’.

Participants who indicated that they had used nitrous oxide answered another subsequent question: “Do you think you will use nitrous oxide again?” Answer options were ‘definitely’, ‘maybe’, and ‘never again’.

#### Predisposing factors

Predisposing factors of the integrated change model were used to select relevant factors [24]. This model considers biological factors, social & cultural factors, psychological factors, and behavioral factors as factors preceding behavior.

#### Biological factors and social-cultural factors

Characteristics such as age (in years), gender (boy vs. girl), ethnic background (Dutch vs. non-Dutch), school level (pre-vocational education vs. senior secondary & pre-university education), and living situation (living with both parents vs. not living with both parents) were entered as biological and social-cultural factors. Ethnic background was classified as Dutch or non-Dutch, by following the definition of Statistics Netherlands; adolescents with at least one parent born outside the Netherlands were classified as non-Dutch [25]. With regard to school level; in the Netherlands, three general levels of secondary education can be distinguished. The lowest level is pre-vocational education that lasts four years and prepares for intermediate vocational education. The next level is senior secondary education that lasts five years and prepares for the university of applied sciences. Lastly, the highest level is pre-university education that lasts six years and prepares for the university [26].

#### Psychological factors and health

Internalizing and externalizing difficulties were assessed by the Dutch self-report version of the Strengths and Difficulties Questionnaire (SDQ) [27]. The SDQ consists of 25 items regarding emotional problems, conduct problems, hyperactivity-inattention, peer problems, and prosocial behavior, all scored on a 3-point scale: 0 = ‘not true’, 1 = ‘somewhat true’, and 2 = ‘certainly true’. The SDQ can be divided into two subscales, i.e. an internalizing scale (items on emotional and peer problems) and an externalizing scale (items on conduct problems and hyperactivity-inattention), both ranging from 0–20. A higher score indicates more problems [28].

Mental well-being was assessed by the Warwick-Edinburgh Mental Well-being Scale (WEMWBS) [29]. This scale consists of 14 items assessing elements of mental well-being, such as happiness and sense of purpose in life. Each of the 14 items was answered on a 5-point Likert scale (1 ‘none of the time’ to 5 ‘all of the time’), based on the adolescents’ experiences in the past two weeks. A total score is generated based on the 14 answers, with a minimum total score of 14 and a maximum total score of 70. A higher score indicated a higher level of mental well-being.

Sickness absence was assessed by the question: “How many days in the past four weeks have you been absent from school because you were sick?” (Answer categories ranged from ‘0 days’ to ‘≥ 7 days’) [30]. For analysis purposes, sickness absence in the past four weeks was recoded into ‘0 days’, ‘1 day’, and ‘≥ 2 days’.

#### Behavioral factors

Truancy was assessed by the question: “How many days in the past four weeks have you been absent from school because you were truanting?” (Answer categories ranged from ‘0 days’ to ‘≥ 7 days’) [23]. For analysis purposes, truancy in the past four weeks was dichotomized into ‘0 days’ and ‘≥ 1 day’.

Binge drinking was assessed by the question: “How many times in the past four weeks did you consume 5 or more alcoholic drinks on one occasion in the past four weeks? (for example at a party or in one evening)”; by following the international definition of binge drinking [31]. The response categories ranged from ‘never’ to ‘nine or more times’ and were recoded into ‘not once’, ‘once’, and ‘≥ 2 times’.

Cigarette smoking was assessed by the question: “Did you ever smoke?”(Answer categories ranged from ‘no’ to ‘yes a whole cigarette or more’ and were dichotomized into ‘never’ and ‘≥ 1 time in life’ [23].

Cannabis use was assessed by the question: “Did you ever use weed (marijuana) or hashish?” (Answer categories ranged from ‘never’ to ‘30 days or more’ and were dichotomized into ‘never’ and ‘≥ 1 time in life’ [23].

### Data analysis

Descriptive statistics were used to describe the socio-demographic characteristics of adolescents. Differences between the group that had never used nitrous oxide in their life versus the group that had used nitrous oxide in their life were tested by chi-square tests (for categorical variables) and independent sample t-tests (for continuous variables) (Table 1).

**Table 1:** Socio-demographic characteristics of the study population (N = 555).

Socio-demographic characteristics	Total population N = 555	Never used nitrous oxide n = 466	Used nitrous oxide ≥ 1 time n = 86	*p*-value
**Age in years, mean (SD)**				
Age in years	15.6 (0.83), range 14–18	15.6 (0.82)	15.6 (0.91)	0.701
**Gender, n (%)**				
Male	294 (53.0)	248 (53.2)	44 (51.2)	0.726
Female	261 (47.0)	218 (46.8)	42 (48.8)	
**Ethnic background, n (%)**				
Dutch	399 (71.9)	343 (73.6)	53 (61.6)	**0.027**
Non-Dutch	156 (28.1)	123 (26.4)	33 (38.4)	
**School level, n (%)**				
Pre-vocational education	288 (51.9)	226 (48.5)	61 (70.9)	**<0.001**
Senior secondary & pre-university education	267 (48.1)	240 (51.5)	25 (29.1)	
**Living situation, n (%)**				
With both my parents	379 (68.3)	327 (70.2)	49 (57.0)	**0.023**
Not with both my parents	176 (31.7)	139 (29.8)	37 (43.0)	

Note: bold numbers indicate a statistically significant (p<0.05) difference between the group that never used nitrous oxide versus the group that did use nitrous oxide, calculated using an independent-samples t-test (continuous variables) or a chi-square test (categorical variables). There were zero missing answers on the socio-demographic questions. There were three missing answers on the question regarding the use of nitrous oxide.

Descriptive statistics were used to describe adolescents’ nitrous oxide use in their life in terms of frequency and future preferences (Table 2).

**Table 2:** Recreational nitrous oxide use in the total population (N = 555).

Nitrous oxide use	N (%)
**In whole life [3]**	
Never	466 (84.4)
1 time	35 (6.3)
2 times	17 (3.1)
3 times	13 (2.4)
4–10 times	14 (2.5)
≥ 11 times	7 (1.3)
**For the participants who did not use nitrous oxide: Do you think you will ever use nitrous oxide? [89]**	
Definitely	26 (5.6)
Maybe	89 (19.1)
Probably not	67 (14.4)
Definitely not	200 (42.9)
I do not know	84 (18.0)
**For the participants who did use nitrous oxide before: Do you think you will use nitrous oxide again? [469]**	
Definitely	45 (52.3)
Maybe	35 (40.7)
Never again	6 (7.0)

Note: [number of missing answers].

To examine associations of socio-demographic characteristics, internalizing and externalizing problems, mental well-being, sickness absence from school, truancy, and substance use on the one hand and lifetime nitrous oxide use on the other hand, ordinal logistic regression analyses were performed. Nitrous oxide use was entered as an ordinal outcome variable ranging from never used, used one time, and used two times or more. All the other before mentioned factors were entered as predictor variables. We present the univariable and multivariable models (Table 3). To identify if multicollinearity between predictors existed, the variance inflation factors (VIFs) were explored. All VIFs were lower than 2, suggesting weak multicollinearity between predictors [32]. Odds ratios (ORs) and 95% confidence intervals (CIs) were estimated. The estimated odds ratios represent the multiplicative change in the odds for an adolescent to be allocated to a higher lifetime nitrous oxide user category when they would have scored one point higher on a predictor variable. We considered a p-value of 0.05 or lower to be statistically significant. Furthermore, Bonferroni correction was considered to correct for multiple comparisons (*p* 0.05/13 = 0.004) and information on this is presented in Table 3 and discussed in the discussion.

**Table 3:** Results of the univariable and multivariable ordinal logistic regression analyses evaluating associations of biological factors and social-cultural factors, psychological factors and health, and behavioral factors with recreational nitrous oxide use.

	Univariable model^a^	Multivariable model^b^
Biological factors and social-cultural factors	OR (95% CI)*	OR (95% CI)*
Age (in years)	0.97 (0.73; 1.27)	0.90 (0.65; 1.24)
Gender		
Male	Ref.	Ref.
Female	1.06 (0.67; 1.67)	0.87 (0.50; 1.51)
Ethnic background		
Dutch	Ref.	Ref.
Non-Dutch	**1.74 (1.08; 2.80)**	**2.10 (1.22; 3.61)**
School level		
Senior secondary and pre-university	Ref.	Ref.
Pre-vocational	**2.66 (1.62; 4.38)**	**1.88 (1.06; 3.34)**
Living situation		
With both parents	Ref.	Ref.
Not with both parents	**1.82 (1.14; 2.91)**	1.45 (0.85; 2.46)
**Psychological factors and health**		
Internalizing problems (range 0–20)^c^	**1.10 (1.03; 1.19)**	1.04 (0.94; 1.15)
Externalizing problems (range 0–20)^c^	**1.20 (1.12; 1.29)**	**1.10 (1.01; 1.20)**
Mental wellbeing (range 14–70)^d^	**0.96 (0.93; 0.99)**	1.00 (0.97; 1.03)
Sickness absence from school		
0 days/4 weeks	Ref.	Ref.
1 day/4 weeks	1.32 (0.72; 2.40)	1.20 (0.62; 2.32)
≥ 2 days/4 weeks	1.69 (0.99; 2.90)	1.11 (0.60; 2.04)
**Behavioral factors**		
Truancy		
0 days/4 weeks	Ref.	Ref.
≥ 1 day/4 weeks	**4.04 (1.85; 8.83)**	1.60 (0.65; 3.96)
Binge drinking^e^		
0 times/4 weeks	Ref.	Ref.
1 time/4 weeks	1.84 (0.84; 4.01)	1.25 (0.53; 2.97)
≥ 2 times/4 weeks	**4.77 (2.79; 8.16)**	**2.49 (1.25; 4.94)**
Lifetime cigarette smoking		
Never	Ref.	Ref.
≥ 1 time	**4.22 (2.63; 6.79)**	1.68 (0.86; 3.25)
Lifetime cannabis use		
Never	Ref.	Ref.
≥ 1 time	**4.05 (2.45; 6.71)**	**1.98 (1.03; 3.79)**

Note: bold numbers indicate a statistically significant (*p*<0.05) association. Nitrous oxide use was entered as an ordinal variable ranging from never used, used one time, used ≥ two times.

*Odds ratio (OR) and 95% confidence interval (95% CI) from ordinal logistic regression analyses. Missing items: age [0], gender [0], ethnic background [0], living situation [0], internalizing problems [4], externalizing problems [4], mental well-being [0], sickness absence [0], truancy [0], binge drinking [2], lifetime cigarette smoking [1], lifetime cannabis use [3].

^a^ The predictor variables were entered separately in the univariable model.

^b^ The predictor variables were entered simultaneously in the multivariable model.

^c^ As measured with the Strengths and Difficulties Questionnaire (SDQ).

^d^ As measured with the Warwick-Edinburgh Mental Well-being Scale (WEMWBS).

^e^ Binge drinking was defined as consuming 5 or more alcoholic drinks on one occasion.

The intracluster correlation coefficient was calculated to consider the potential variance in nitrous oxide use explained by the clustering of regions. The estimated coefficient was 0.04; therefore, no adjustments for region were performed in subsequent analyses.

Logistic regression analysis were applied to analyze whether participants who responded that they will definitely use nitrous oxide again differed from participants who responded that they would maybe use again, by using the predictor variables from the previous multivariable model.

All analyses were performed using SPSS version 25 (IBM Corp. Released 2017. IBM SPSS Statistics for Windows, Version 25.0. Armonk, NY: IBM Corp.).

## Results

### Participant characteristics

The study population was on average 15.6 years old (SD = 0.83, range 14–18), 47.0% were female and 71.9% were classified as Dutch (Table 1). Of the 552 participants who answered questions about their nitrous oxide use, 86 (15.6%) had used nitrous oxide at least once in their life.

Table 2 presents the descriptive statistics of nitrous oxide use in the total study population. A total of 466 participants had never used nitrous oxide in their life and a total of 86 participants had used nitrous oxide in their life. Furthermore, 35/86 participants had used nitrous oxide once, 51/86 participants had used it twice or more. The largest proportion of participants who had never used nitrous oxide indicated they were sure they would never use it in the future (n = 200/466). The largest proportion of participants who had used nitrous oxide before indicated they would definitely use it again (n = 45/86).

Logistic regression analysis showed that participants who responded they will definitely use again were more often of non-Dutch ethnic background (OR = 5.53), or reported more often binge drinking (OR = 8.01 and 96.57) and cannabis use (OR = 10.37), compared to participants who responded that they would maybe use again.

### Results of the regression analyses

Table 3 shows the association of biological factors and socio-cultural factors, psychological factors and health, and behavioral factors with lifetime nitrous oxide use, as assessed with ordinal logistic regression analyses. In the multivariable model, participants classified as non-Dutch were at risk of having a higher category of nitrous oxide use (OR = 2.10, 95% CI 1.22; 3.61), as were participants who attend pre-vocational education (OR = 1.88, 95% CI 1.06; 3.34). Participants with a higher score on the externalizing SDQ scale were also at risk of having a higher category of nitrous oxide use (OR = 1.10, 95% CI 1.01; 1.20), as were participants who indicated they had been binge drinking twice or more in the past four weeks OR = 2.49, 95% CI 1.25; 4.94), and participants who used cannabis (OR = 1.98, 95% CI 1.03; 3.79).

## Discussion

This study investigated the association of socio-demographic characteristics, internalizing and externalizing problems, mental well-being, sickness absence from school, truancy, and substance use with the frequency of lifetime nitrous oxide use among adolescents (15.6 years). Our results indicate a lifetime nitrous oxide use prevalence of 15.6% among the studied population. Especially a non-Dutch ethnic background, a pre-vocational education school level, externalizing problems, frequent binge drinking, and cannabis use were significantly (*p*<0.05) associated with increased lifetime use of nitrous oxide in the multivariable regression model. If we apply Bonferroni correction for multiple testing, none of the variables in the multivariable model showed a significant association with nitrous oxide use.

The lifetime prevalence of nitrous oxide of 15.6% in our study is comparable with the prevalence in the Health Behavior in School-aged Children study of 2017, where 13.9% of 15-year-olds and 16.9% of 16-year-olds reported lifetime nitrous oxide use [4]. This prevalence is slightly higher than the reported prevalence in a national representative study in 2015 of 10.6% among 15-year-olds and 14.1% among 16-year-olds [3]; lifetime nitrous oxide use among 15-16-year-olds might have increased. Participants also indicated their future preferences regarding using nitrous oxide. Approximately 90% of previous users and 24% of participants who had never used before indicated they would definitely or maybe like to use nitrous oxide in the future (again). Comparing users who would definitely re-use with users who would maybe re-use indicated that participants with a non-Dutch ethnic background, who had been binge drinking and who had used cannabis had a higher odds ratio to fall into the category of definitely using again. We recommend studying this in more detail in the future, for example by applying a quantitative study design with a large number of participants to improve the external validity of the results. Also in-depth qualitative designs are recommended to study the reasons for adolescents to re-use nitrous oxide.

The high number of participants that would like to (re-)use nitrous oxide indicates that the image that participants have of nitrous oxide is relatively positive. This could partly be explained by the fact that at the time of this study, nitrous oxide could by legally obtained under the Dutch commodities law, possibly contributing to the “innocent” image of nitrous oxide. Previous research in England among young adults using nitrous oxide, indicated that that there is *‘a lack of concern with side effects*, *coupled to a willingness to partake’* [33]. Previous research in the Netherlands among professionals who are involved with nitrous oxide users, suggested similar motivations to re-use nitrous oxide in under aged secondary school students [34]. We therefore recommend that the prevention of nitrous oxide should start in early adolescence.A study by Bennett et al. reported that early use (i.e. before age 18) of either inhalants or marijuana substantially increased risk of frequent drinking, binge drinking, smoking, illicit drug use, and substance-related consequences during the college years [35]. Furthermore, a previous study found that adolescents who once have used nitrite inhalants also tend to use, or continue to use, other substances and progress to drug abuse or dependence [36]. It is unclear whether something similar occurs for nitrous oxide use. We recommend further investigation on this topic to uncover whether nitrous oxide serves as a ‘gateway’ to other drug use.

In our study, participants with a non-Dutch ethnic background had higher odds of increased lifetime nitrous oxide use. This resonates with findings from the Health Behaviour in School-aged Children study among 12-16-year-olds where participants with a non-western migration background had a significantly higher prevalence of lifetime nitrous oxide use than participants who had no migration background [4]. This significant result is not visible in the general adult population in the Netherlands [37]. Nabben et al., based on information from experts, distinguishes a group of beginning nitrous oxide users that is typically formed by under aged secondary school students. These users are relatively often from urban areas and often have a migration background. For this group, nitrous oxide does not have the status of being a drug and is seen as more innocent than alcohol or cannabis. Moreover, using alcohol or cannabis is considered a taboo, for example for religious reasons [34]. Other research suggests that ethnic differences in adolescent tobacco, alcohol, and drug use are possibly explained by background and lifestyle factors, such as educational values and religious commitment [38]. We recommend future studies to explore the association between substance use, in particular nitrous oxide, and ethnic background.

Our results indicated that participants who attended pre-vocational education had higher odds of lifetime nitrous oxide use compared with adolescents attending higher educational levels. This finding is confirmed by previous studies showing that lower Grade Point Average in adolescence was associated with alcohol and illicit drug use [39,40].

The association we found between externalizing problems and increased lifetime use of nitrous oxide concurs with previous research where associations were found between externalizing behavior problems and the use of alcohol and drugs among adolescents [41,42]. Externalizing behavior consists of outward behavior, such as impulsive and deviant behavior that may be closely linked to risk health behaviors, for instance, drug use [41].

Further, our findings suggest an association of frequent binge drinking, cannabis use, and increased lifetime nitrous oxide use. The multivariable model also showed a large decrease in the odds ratio of smoking and cannabis use compared to the univariable model. Exploratory analysis showed that a large decrease in the odds ratio of smoking occurs when adding binge drinking or cannabis use to the model. A large decrease in the odds ratio of cannabis use occurs when adding binge drinking or smoking to the model (results of these exploratory analysis can be found in S2 Table). Co-occurrence of risk behaviors has been reported in other studies where clustering of alcohol misuse, smoking, and nitrite inhalants or illicit drug use was found among adolescents and young adults [36,43]. The combination of heavy alcohol drinking and nitrous oxide use is suggested to be a dangerous combination because heavy alcohol drinking may disrupt the stimulus to breathe, which could lead to a deficit in oxygen if nitrous oxide is inhaled [44].

We could not demonstrate a link between school attendance problems (i.e. sickness absence or truancy) and nitrous oxide use in the multivariable model. Previous research did find an association between attendance problems and substance use other than nitrous oxide [39]. One explanation can be found in the result of exploratory analysis where the significant association between truancy and nitrous oxide use in the univariable model disappears after adding binge drinking to the model (results of these exploratory analysis can be found in S3 Table). This suggests that binge drinking and truancy are related. Another explanation might be that the use of nitrous oxide is particularly high at parties and festivals [2,37], therefore, possibly does not interfere with school attendance.

We highlight the potential for interventions to target risk behaviors, such as nitrous oxide use, binge drinking, and cannabis use, preferably starting in early adolescence, to protect their developing brains before the risk behaviors are occurring. School-based interventions using approaches of social competence and social influence have been shown protective of drugs and cannabis use: we recommend to study whether this approach can be applied to promote a broad healthy lifestyle, including avoiding the risks of nitrous oxide use [45].

This study has several limitations that warrant consideration when interpreting the results. First, we might have missed some factors relevant to nitrous oxide use that were not available in our study, such as criminal behaviors. Second, for the outcome variable, we depended on a self-reported question asking about the lifetime use of nitrous oxide. We did not take into account how many balloons filled with nitrous oxide were inhaled at one occasion. The highest number of balloons inhaled at one occasion has been reported to be associated with accidental injury [1]. Third, we could not detect certain trends over time as the questionnaire was analyzed cross-sectionally. A study with multiple follow-up measures is needed to draw conclusions on the direction of the associations. Fourth, if a Bonferroni correction for multiple testing was applied, the significant results in the multivariable model all disappeared. Therefore non-corrected results should be interpreted with caution. However, a Bonferroni correction can be considered strict and might increase the change of observing false negative findings [46]. Lastly, we included adolescents from different areas in the Netherlands. However, the sample size was relatively small. We recommend future research to include a large sample of adolescents from several countries to increase the external generalizability of the results. This was one of the first studies that explored what factors were associated with lifetime nitrous oxide use among adolescents. Therefore, it is necessary that future research investigates whether our findings can be replicated.

## Conclusions

This study assessed the association of adolescents’ lifetime nitrous oxide use with socio-demographic characteristics, internalizing and externalizing problems, mental well-being, sickness absence from school, truancy, and substance use. A non-Dutch ethnic background, attending pre-vocational education, externalizing problems, binge drinking more than once in the past month, and cannabis use were associated with increased lifetime use of nitrous oxide. Our findings give implications for policy and practice to address drug use and in particular nitrous oxide use as an increasingly popular drug among adolescents and to promote healthy adolescent’ lifestyles from an early age onwards.

## Supporting information

S1 Table Overview of the questionnaire. (DOCX)

S2 Table Results of the exploratory analyses evaluating the change in odds ratio when adding predictor variables to the univariable models of cigarette smoking, and cannabis use. (DOCX)

S3 Table Results of the exploratory analyses evaluating the change in odds ratio when adding predictor variables to the univariable models of truancy. (DOCX)
