# Supplementary material for: Associations of socio-demographic characteristics, well-being, school absenteeism, and substance use with recreational nitrous oxide use among adolescents: A cross-sectional study
Source: PLoS One. 2021 Feb 18;16(2):e0247230. doi: 10.1371/journal.pone.0247230 (PMC7891713; doi:10.1371/journal.pone.0247230)
Supplement: S1 Table — (DOCX) [file pone.0247230.s001.docx]

**Supplementary Table 1.** Overview of the questionnaire.

| **Predictor or outcome** | **Official instrument** |
| --- | --- |
| Internalizing problems | The Strengths and difficulties questionnaire |
| Externalizing problems | The Strengths and difficulties questionnaire |
| Mental well-being | Warwick Edinburgh Mental Well-being Scale |
| Sickness absence | Derived from the Health Behavior in School-aged Children study |
| Truancy | Derived from the Health Behavior in School-aged Children study |
| Binge drinking | Derived from the Health Behavior in School-aged Children study |
| Cigarette smoking | Derived from the Health Behavior in School-aged Children study |
| Cannabis use | Derived from the Health Behavior in School-aged Children study |
| Nitrous oxide use | Derived from the Health Behavior in School-aged Children study |
