# Supplementary material for: Associations of socio-demographic characteristics, well-being, school absenteeism, and substance use with recreational nitrous oxide use among adolescents: A cross-sectional study
Source: PLoS One. 2021 Feb 18;16(2):e0247230. doi: 10.1371/journal.pone.0247230 (PMC7891713; doi:10.1371/journal.pone.0247230)
Supplement: S2 Table — (DOCX) [file pone.0247230.s002.docx]

| **Supplementary Table 2.** Results of the exploratory analyses evaluating the change in odds ratio when adding predictor variables to the univariable models of cigarette smoking, and cannabis use. | | |
| --- | --- | --- |
| **Factors added to model separately** | **Model 1^a^**  **Cigarette smoking** | **Model 2^b^**  **Cannabis use** |
|  | **OR (95% CI)*** | **OR (95% CI)*** |
| **Biological factors and social-cultural factors** |  |  |
| Age (in years) | 4.25 (2.64; 6.85) | 4.22 (2.53; 7.04) |
| Gender | 4.22 (2.62; 6.79) | 4.26 (2.55; 7.12) |
| Ethnic background | 4.30 (2.67; 6.94) | 4.26 (2.55; 7.09) |
| School level | 3.97 (2.45; 6.41) | 3.99 (2.39; 6.66) |
| Living situation | 4.11 (2.55; 6.63) | 3.98 (2.40; 6.61) |
| **Psychological factors and health** |  |  |
| Internalizing problems (range 0-20)^c^ | 4.22 (2.62; 6.81) | 4.23 (2.54; 7.05) |
| Externalizing problems (range 0-20)^c^ | 3.30 (2.01; 5.41) | 3.23 (1.92; 5.44) |
| Mental wellbeing (range 14-70)^d^ | 3.94 (2.44; 6.37) | 3.99 (2.40; 6.63) |
| Sickness absence from school | 4.10 (2.54; 6.61) | 4.07 (2.45; 6.77) |
| **Behavioral factors** |  |  |
| Truancy | 3.85 (2.37; 6.25) | 3.67 (2.20; 6.14) |
| Binge drinking^e^ | 2.99 (1.71; 5.20) | 2.77 (1.60; 4.79) |
| Lifetime cigarette smoking | NA | 2.05 (1.12; 3.74) |
| Lifetime cannabis use | 3.01 (1.71; 5.29) | NA |

Note: Nitrous oxide use was entered as an ordinal variable ranging from never used, used one time, used ≥ two times.

^*^Odds ratio (OR) and 95% confidence interval (95% CI) from ordinal logistic regression analyses.

^a^ Model 1 represented the univariable model of cigarette smoking and nitrous oxide use. The original odds ratio was 4.22 (2.63; 6.79).

^b^ Model 2 represented the univariable model of cannabis use and nitrous oxide use. The original odds ratio was 4.05 (2.45; 6.71).

^c^ As measured with the Strengths and Difficulties Questionnaire (SDQ).

^d^ As measured with the Warwick-Edinburgh Mental Well-being Scale (WEMWBS).

^e^ Binge drinking was defined as consuming 5 or more alcoholic drinks on one occasion.
