# Supplementary material for: Associations of socio-demographic characteristics, well-being, school absenteeism, and substance use with recreational nitrous oxide use among adolescents: A cross-sectional study
Source: PLoS One. 2021 Feb 18;16(2):e0247230. doi: 10.1371/journal.pone.0247230 (PMC7891713; doi:10.1371/journal.pone.0247230)
Supplement: S3 Table — (DOCX) [file pone.0247230.s003.docx]

| **Supplementary Table 3.** Results of the exploratory analyses evaluating the change in odds ratio when adding predictor variables to the univariable models of truancy. | |
| --- | --- |
| **Factors added to model separately** | **Model 1^a^**  **Truancy** |
|  | **OR (95% CI)*** |
| **Biological factors and social-cultural factors** |  |
| Age (in years) | 4.21 (1.90; 9.32) |
| Gender | 4.06 (1.85; 8.88) |
| Ethnic background | 4.13 (1.89; 9.06) |
| School level | 4.15 (1.86; 9.23) |
| Living situation | 3.93 (1.78; 8.65) |
| **Psychological factors and health** |  |
| Internalizing problems (range 0-20)^b^ | 3.45 (1.55; 7.68) |
| Externalizing problems (range 0-20)^b^ | 2.84 (1.26; 6.37) |
| Mental wellbeing (range 14-70)^c^ | 3.73 (1.69; 8.23) |
| Sickness absence from school | 3.59 (1.60; 8.06) |
| **Behavioral factors** |  |
| Truancy | NA |
| Binge drinking^d^ | 2.29 (0.99; 5.30) |
| Lifetime cigarette smoking | 2.44 (1.08; 5.52) |
| Lifetime cannabis use | 2.91 (1.29; 6.60) |

Note: Nitrous oxide use was entered as an ordinal variable ranging from never used, used one time, used ≥ two times.

^*^Odds ratio (OR) and 95% confidence interval (95% CI) from ordinal logistic regression analyses.

^a^ Model 1 represented the univariable model of truancy and nitrous oxide use. The original odds ratio was 4.04 (1.85; 8.83).

^b^ As measured with the Strengths and Difficulties Questionnaire (SDQ).

^c^ As measured with the Warwick-Edinburgh Mental Well-being Scale (WEMWBS).

^d^ Binge drinking was defined as consuming 5 or more alcoholic drinks on one occasion.
